# Functional role of vitronectin in breast cancer

**DOI:** 10.1371/journal.pone.0242141

**Published:** 2020-11-19

**Authors:** Alakesh Bera, Madhan Subramanian, John Karaian, Michael Eklund, Surya Radhakrishnan, Nahbuma Gana, Stephen Rothwell, Harvey Pollard, Hai Hu, Craig D. Shriver, Meera Srivastava

**Affiliations:** 1 Department of Anatomy, Physiology and Genetics, Uniformed Services University of the Health Sciences (USUHS), Bethesda, Maryland, United States of America; 2 Chan Soon-Shiong Institute of Molecular Medicine, Windber, Pennsylvania, United States of America; 3 Murtha Cancer Center, Walter Reed National Military Medical Center, Bethesda, Maryland, United States of America; University of Hawai’i at Manoa, UNITED STATES

## Abstract

Breast Cancer is the most common form of cancer in women worldwide, impacting nearly 2.1 million women each year. Identification of new biomarkers could be key for early diagnosis and detection. Vitronectin, a glycoprotein that is abundantly found in serum, extracellular matrix, and bone, binds to integrin αvβ3, and promotes cell adhesion and migration. Current studies indicate that patients with amplified vitronectin levels have lower survival rates than patients without amplified vitronectin levels. In this study, we focused on the role of vitronectin in breast cancer survival and its functional role as a non-invasive biomarker for early stage and stage specific breast cancer detection. To confirm that the expression of vitronectin is amplified in breast cancer, a total of 240 serum samples (n = 240), 200 from breast cancer patients and 40 controls were analyzed using the Reverse Phase Protein Array (RPPA) technique. Of the 240 samples, 120 samples were of African American (AA) descent, while the other 120 were of White American (WA) descent. Data indicated that there were some possible racial disparities in vitronectin levels and, differences also seen in the recurrent patient samples. Next, we tried to uncover the underlying mechanism which plays a critical role in vitronectin expression. The cellular data from four different breast cancer cell lines- MCF7, MDA-MB-231, MDA-MB-468, and HCC1599 indicated that the PI3K/AKT axis is modulating the expression of vitronectin. We believe that vitronectin concentration levels are involved and connected to the metastasis of breast cancer in certain patients, specifically based on recurrence or ethnicity, which is detrimental for poor prognosis. Therefore, in this current study we showed that the serum vitronectin levels could be an early marker for the breast cancer survival and we also determine the cellular signaling factors which modulate the expression and concentration of vitronectin.

## Introduction

Breast Cancer (BC) is the most common form of cancer effecting women worldwide [1]. According to the World Health Organization, nearly 2.1 million women are diagnosed with this disease annually, and in 2019 about 650,000 women died of breast cancer [1, 2]. Due to improvements in diagnostic techniques and treatments over the last decade, survival rates of BC patients surviving greater than 5 years has improved to 89.9%; however, survival rates still widely vary within different stages of breast cancer. Survival rates of BC patients at the localized stage is 98.8%, at the regional stage is 85.5%, and at a distant stage is 27.4% [1]. Even after successful treatment of BC by lumpectomy, radiation therapy, or mastectomy, the risk of local or distant recurrence is still prevalent in many patients [3]. The survival rate of recurrent BC patients is about 42% [4]. The identification of a new biomarker for breast cancer recurrence could vastly improve the survival rates of BC patients.

Vitronectin is a multifunctional glycoprotein that is highly present in serum, extracellular matrix, and bone [5, 6]. The amino terminal of vitronectin is made of 44 amino acids that are identical to that of somatomedin B (SMB), which is a serum factor rich in cysteine and involved in the binding of plasminogen activator inhibitor-1 (PAI-1) [6]. The amino acid sequence that follows the SMB sequence consists of Arginine-Glycine-Aspartic acid (RGD) sequence, which is responsible for mediating the attachment and spreading of cells to the extracellular matrix via integrin receptors [7–11]. The carboxyl terminal of vitronectin contains a group basic amino acids that accommodates the plasminogen binding site as well as more residues involved in binding of PAI-1 [6, 7, 11–14].

Integrins are proteins that consist both alpha (α) and beta (β) subunits that can form 24 different combinations of transmembrane heterodimers when working together and play a critical role in different cancers [10, 15, 16]. Integrin’s role as an adhesion receptor is critical for extracellular ligands to transduce signals into the cell [15]. Integrin is also found to be bidirectional, sending signals into or out of the cell. Integrins can directly bind to components of the extracellular matrix (ECM) and provide the adhesion necessary for cell motility and invasion [15]. Vitronectin is typically anchored to the ECM due to its collagen and heparin binding sites. This allows vitronectin to interact with some integrins such as αvβ3, αvβ5, αvβ1, αIIbβ3, αvβ6, and αvβ8 to promote cell adhesion, migration and spreading [6]. Interaction between vitronectin and Integrin αvβ3, also known as the vitronectin receptor, could be key to understanding vitronectin’s role in cancer [8]. Integrin αvβ3 plays a crucial role in tumor progression such as angiogenesis in cancer cells and in neovascularization of tumors and in bone metastasis of breast cancer [9, 15, 17].

In this current study, we examined the functional role of vitronectin in BC. We focused on vitronectin expression levels in various cancers, and found that BC cell lines had higher levels of amplified copy number of vitronectin than any other cancer type. Next, based on that correlation between vitronectin and BC, we analyzed 200 serum BC patient samples and 40 controls. Of the 240 samples, 120 were of AA descent and the other 120 were of WA descent. Analysis of the serum samples revealed that there were significant racial disparities in serum vitronectin levels in BC patients. This disparity in vitronectin serum levels was especially seen in the recurrent patient samples, where they were retested.

## Materials and methods

### Ethics statement

The study was approved by the joint institutional review board (IRB) of Walter Reed National Military Medical Center (WRNMMC) and the Uniformed Services University, Bethesda, MD. There were no subjects (patients) included lower than age of 18 for this research and, all patients recruited for the study provided written informed consent for the research.

### Patient enrollment and serum collection

The details protocol and procedures were described earlier [18, 19]. Briefly, all patients were enrolled into the Clinical Breast Care Project at Murtha Cancer Center at the WRNMMC, Bethesda following Institutional Review Board approved. A total of 240 patient cases (n = 240) were selected, composed of controls and all immunohistochemistry-based subtypes—luminal, basal-like, human epidermal HER2 overexpressing, and normal-like (Table 1). These were also subdivided by pre- and post-menopausal statuses and by race. We had 21 serum samples from patients, which showed recurrence after 4 to 7 years of disease-free survival (details in Table 1).

**Table 1:** Displays information on the 240 serum samples used during RPPA analysis.

Patient/Tumor Characteristics	AA Samples (n = 120)	WA samples (n = 120)
Control, Pre-menopausal	10	10
Control, Post-menopausal	10	10
TN, Pre-	15	10
TN, Post-	14	10
HER2+, Pre-	6	10
HER2+, Post-	1	10
LA, Pre-	10	10
LA, Post-	8	10
LB1, Pre-	15	10
LB1, Post-	22	10
LB2, Pre-	5	10
LB2, Post-	4	10

The Table breaks down samples into various patients with tumor sub-types characteristics. Abbreviation used: AA-African American, WA-White American, TN, triple-negative subtype; Her2, human epidermal growth factor receptor 2 subtype; LA, Luminal A subtype; LB1, Luminal B1 subtype; LB2, Luminal B2 subtype.

### Cells

Four breast cancer cell lines were chosen for the experiment and were purchased from American Type Cell Culture (ATCC): MCF-7 (HTB-22), MDA-MB-231 (HTB-266), MDA-MB-468 (HTB-132), and HCC-1599 (CRL-2331). The cells were incubated at 37°C at 5% CO_2_ and were maintained in Minimum Essential Media supplemented with 10% FBS and antibiotics (Penicillin/Streptomyocin). All cell lines were Mycoplasma free. We tested Mycoplasma in every 28-day culture test by using ThermoFisher MycoSEQTM Mycoplasma detection kit.

### Reverse Phase Protein Array (RPPA) printing

Serum samples were thawed on ice and well mixed prior to aliquoting to an initial dilution of 1:20 in printing buffer. Additional dilutions were made manually in a serial, two-fold manner for a total of 7 dilution points. Samples were then transferred to 384-well source plates using a Janus Liquid Handling system (Model AJL8M01, PerkinElmer, Waltham, MA). All dilution and source plates were sealed and kept at 4°C until printing was started. Samples were printed to nitrocellulose slides (ONCYTE^®^ NOVA Nitrocellulose Film Slides, Arrayjet, UK) using an Aushon 2470 Microarray Printer (Aushon, Billerica, MA). Each sample was deposited twice in succession at room temperature and maximum humidity. All 240 samples were printed randomly distributed across two arrays in side-by-side technical duplicates.

### RPPA staining, imaging, and analysis

Once the samples were printed onto the slide, the slides were stained with the primary and secondary antibodies. Prior to staining the slides with the primary antibody, the slides were washed with a blocking buffer made of 5% BSA for 1 hour at room temperature. The slides were then washed with wash buffer 2 (WB2), which is 1x Tris-buffered saline, two times at room temperature. After washing, the slides were stained with the vitronectin primary antibody, placed in a humidity chamber, and placed in a -4 °C fridge to incubate overnight. After slides are done incubating, they were washed with wash buffer 1 (WB1), which is 1x Tris-buffered saline with 1% tween, 3 times at room temperature. Then, slides are incubated in dark, covered from light, with the secondary antibody for 1 hour at room temperature. After this, slides are washed with WB1 3 times at room temperature, and then WB2 3 times at room temperature. Then, slides are quickly air dried and were placed into the scanner.

The slides were scanned using the InnoScan (710) at a wavelength of 675 nm, and using the XDR (extended dynamic range) feature. Once the slides were scanned, the image analysis software, Mapix, was used to extract fluorescence intensity values from the raw images produced by the scanner.

### Data analysis and statistical models

The resulting spot quantification were then pre-processed and analyzed using R (v. 3.6.1). Arrays were paired between primary + secondary antibody slides (primary slides) and their respective secondary antibody only (secondary slides) slides. Local background correction was performed for each spot separately, subtracting background signal from foreground signal. Any negative net signal was removed (primary slides) or converted to 0 (secondary slides). Spots were then controlled for quality by removing any spots with a signal to noise ratio (SNR) less than 4 for primary slides, and less than 2 for secondary slides. Corrections for non-specific signaling were made by subtracting net signals of the secondary slides from their respective primary slides on a spot-by-spot basis. Net signal was then normalized by the median slide intensity and all values were log2 transformed.

Sample values were determined by extrapolating a zero dilution point by linear regression of the normalized signal versus dilution steps. Linear fits were then evaluated for goodness of fit by manually inspecting all regressions with an R^2^ < 0.50. Spots significantly outside the 95% confidence interval, or which did not appear to follow any discernible linear trend were removed. Samples with less than half of their spots were removed from the data set and not considered for the remaining analyses.

### Capillary electrophoresis and immunoassay

Capillary electrophoresis and immunoassay or SimpleWestern analyses were performed using the Wes machine (ProteinSimple, Biotechne, San Jose, CA) according to the manufacturer’s protocol. Protein Simple: https://www.proteinsimple.com/wes.html. Briefly, 1 μg total lysate samples were mixed with a master mix (ProteinSimple) to a final concentration of 1x sample buffer, 1x fluorescent molecular weight markers, and 40 mM dithiothreitol (DTT), and then heated at 70°C for 6 min. The samples, blocking reagent, primary antibodies, HRP-conjugated secondary antibodies, chemiluminescent substrate, and separation and stacking matrices were also dispensed to designated wells in a pre-designed plate (ProteinSimple). After plate loading, the separation electrophoresis and immune-detection steps took place in the capillary system and were fully automated. Simple Western analysis was carried out at room temperature, and instrument default settings with high dynamic range (HDR) for the chemiluminescent detection step. The vitronectin primary antibody (RD systems, MAB2349), PI3K (Cell Signaling Technology, 4292), P-AKT (Cell Signaling Technology, #9271), AKT (Cell Signaling Technology, #9272) were diluted with antibody diluent (ProteinSimple). The digital image was analyzed with Compass software (ProteinSimple), and the quantified data of the detected protein was reported as molecular weight. Quantified data extracted from the Compass software included the area of peaks produced at the expected molecular weight of the protein. This area value was taken for each protein in each cell line and then normalized to the housekeeping genes, GAPDH and B-actin, in each cell line. The GAPDH and B-actin area values were averaged, and then proteins were normalized based on this average value.

### Database, bioinformatics analysis, and statistics

RPPA data were analyzed for statistical significance using a three-way ANOVA between IHC groups, ethnicity menopausal status and their interactions. Since recurrence is only known in a small subset of the total samples, a separate three-way ANOVA was performed between those samples with a known recurrence status plus control samples. Two-tailed t-tests were performed between specific groups for variables found to be, or approaching statistical significance. To calculate receiver operating characteristic (ROC) curve, we used GraphPad Prism 7.05.

## Results

### Vitronectin levels in various cancers

**Fig 1:**
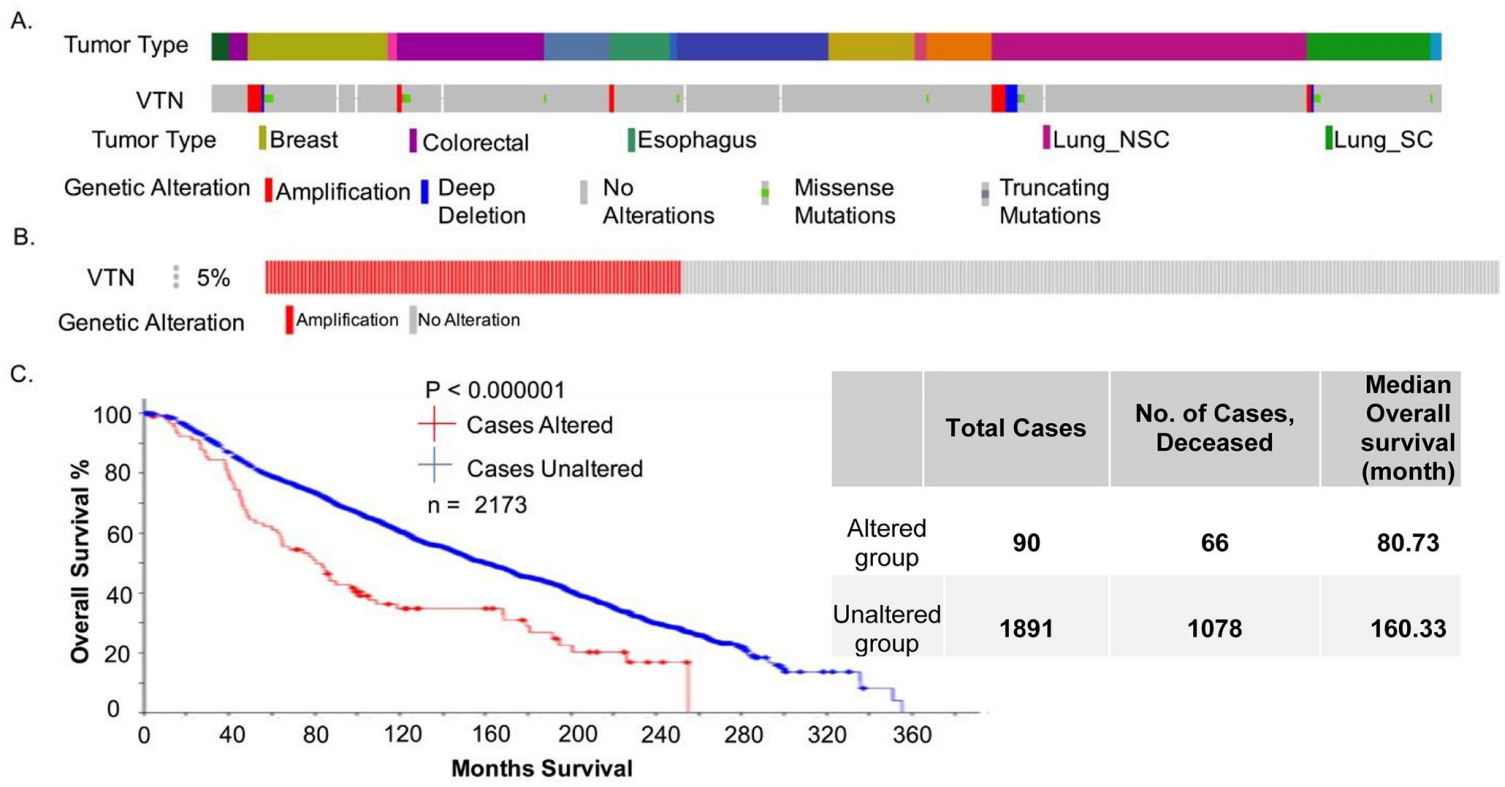
Copy number alteration and survival data. **A**. Illustrates percentage of VTN gene altered in a sample of 881 tumor specimens from CCLE database. Across, all cancer cell lines, only 3% of the VTN is altered by either amplification or deletion of the gene, however, the BC cell lines have a high percentage of VTN copy number amplified. **B**. Data illustrates the percentage of BC tumor specimens that have their VTN copy number amplified in a sample of 2173 BC patients from the METABRIC database. About 5% of these patients had their VTN copy number amplified. **C**. Data indicates the survival rates of BC patients with and without the alteration (CNA, amplification) to the VTN gene from the same study seen in B.

We analyzed data sets from the curated databases of the cancer cell line encyclopedia (CCLE, n = 1739) [20, 21] and Breast Cancer tumor samples (METABRIC study, n = 2173) using cBioportal platform [22–25]. After analyzing the CCLE dataset (Fig 1A), which consisted of 1739 cancer cell lines (over 20 different types of cancer cell-lines), we determined that about 3% of all cases had its vitronectin copy number is amplified. However, when isolating just the breast cancer cell lines, we found that there are more cell lines with vitronectin copy number amplified than in any other cancer cell line (Fig 1A). Next, looking at the METABRIC data set, which consisted of 2173 BC patient samples (n = 2173), we determined 5% of these cases had the vitronectin copy number amplified (Fig 1B.). Using the same dataset, we analyzed the survival rate of individuals with and without the amplification of vitronectin copy number (Fig 1C.), and it was found that the survival rate significantly (p<0.000001) decreased for patients that had this amplification of vitronectin.

### Serum vitronectin concentration levels in breast cancer patients

Based on the findings of genomic data analysis we hypothesized that concentration of vitronectin in serum could be a prognostic marker for breast cancer patients. Next, we analyzed 240 BC patient serum samples to look at the overall concentration levels of vitronectin (Table 2). Of the 240 BC patients, 120 were of AA descent, while the other 120 were of WA descent. Patient samples also represented a wide range of tumor characteristics that define patient’s tumor subtype of BC based off immunohistochemistry (IHC) assays.

**Table 2:** Displays mean fold change and standard error (SE) from the RPPA dataset, comparing race and tumor subtypes to the control group. Data allows us to compare level of vitronectin in serum. The mean fold change is relative to the control population for the same ethnicity.

Group	Mean Fold Change	SE
**Cancer**	0.40	0.10
**Control**	0.00	0.18
**AA Cancer**	0.20	0.16
**AA Control**	0.00	0.25
**WA Cancer**	0.60	0.12
**WA Control**	0.00	0.28
**AA Control**	0.00	0.25
**AA Her2+**	0.98	0.47
**AA LA**	-0.28	0.24
**AA LB1**	0.40	0.30
**AA LB2**	0.87	0.34
**AA TN**	-0.25	0.32
**WA Control**	0.00	0.28
**WA Her2+**	0.91	0.24
**WA LA**	0.02	0.26
**WA LB1**	0.34	0.29
**WA LB2**	0.61	0.28
**WA TN**	0.87	0.28

**Fig 2:**
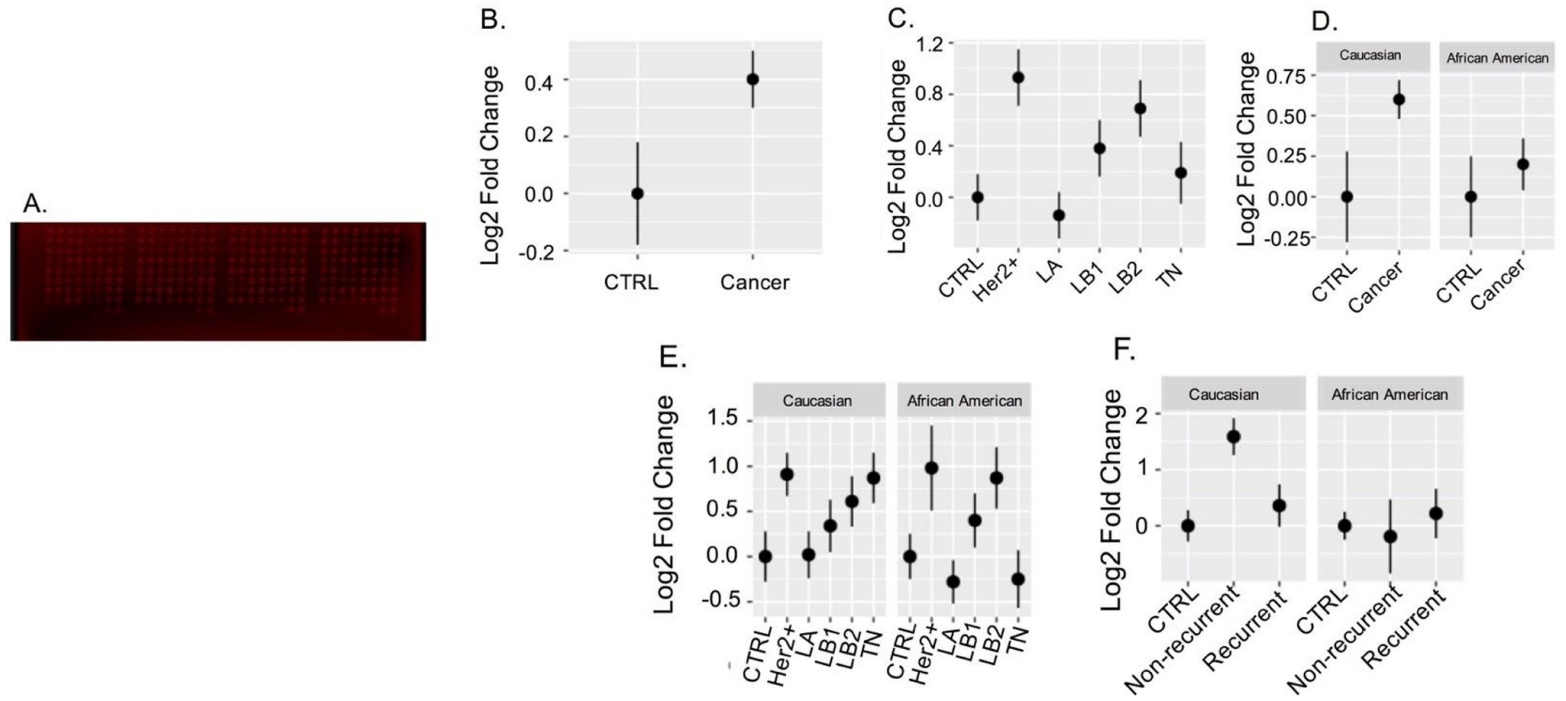
Serum concentration levels of vitronectin in patient samples from RPPA. **A**. Image displays the RPPA dot blot which was stained with the vitronectin antibody. Intensity values were taken from here and then analyzed. **B-F**. Displays comparisons of mean relative values of each group using standard error for error bars. **B**. analyzes overall serum vitronectin levels in control group (n = 40) and cancer group (n = 200). **C**. analyzes serum vitronectin levels (overall) in control groups and compared with the other tumor subtypes. Her2+ and LB2 show significant differences when compared to control group. **D**. compares control and cancer groups by ethnicity, and we find that CA cancer shows a significantly higher vitronectin concentration levels. **E**. we see that it compares vitronectin levels of AA and WA patients by dividing into various tumor characteristics. However, we found that there are significant differences in Her2+ and LB2 subtypes in the AA group when compared to AA controls. **F**. compares recurrent versus non-recurrent patient samples by ethnic groups. We found that there is a significant difference in serum vitronectin levels in non-recurrent CA compared to the control and recurrent groups, while AA samples had similar levels of serum vitronectin in all three groups.

To obtain the vitronectin concentration levels in BC patient samples, we used the RPPA technique as previously explained to look for overall concentration of vitronectin (Fig 2A.). Analysis of the data shows that there is difference in vitronectin concentration levels between control groups versus cancer with a p-value 0.0149 (Fig 2B). Next, we also found that overall vitronectin levels were significantly different between control and Her2+ patients (p = 0.004) and control and LB2 patients (p = 0.049) (Fig 2C). Ethnicity and menopausal status alone were not found to be significant but an interaction between IHC group, ethnicity and menopausal status approached significance (p = 0.057) (Fig 2D–2E) and might be worth exploring with a larger samples size (each group limited to a sample size of ~10 or less). A three-way ANOVA between the recurrence status and control subset found a statistically significant interaction between IHC non-recurrence group and recurrence (p = 0.0206) and IHC group (cancer) and Ethnicity (p = 0.0433) (Fig 2F). A two-tailed t-test found a statistically significant difference between recurrent and non-recurrent WA patients (p = 0.030).

**Fig 3:**
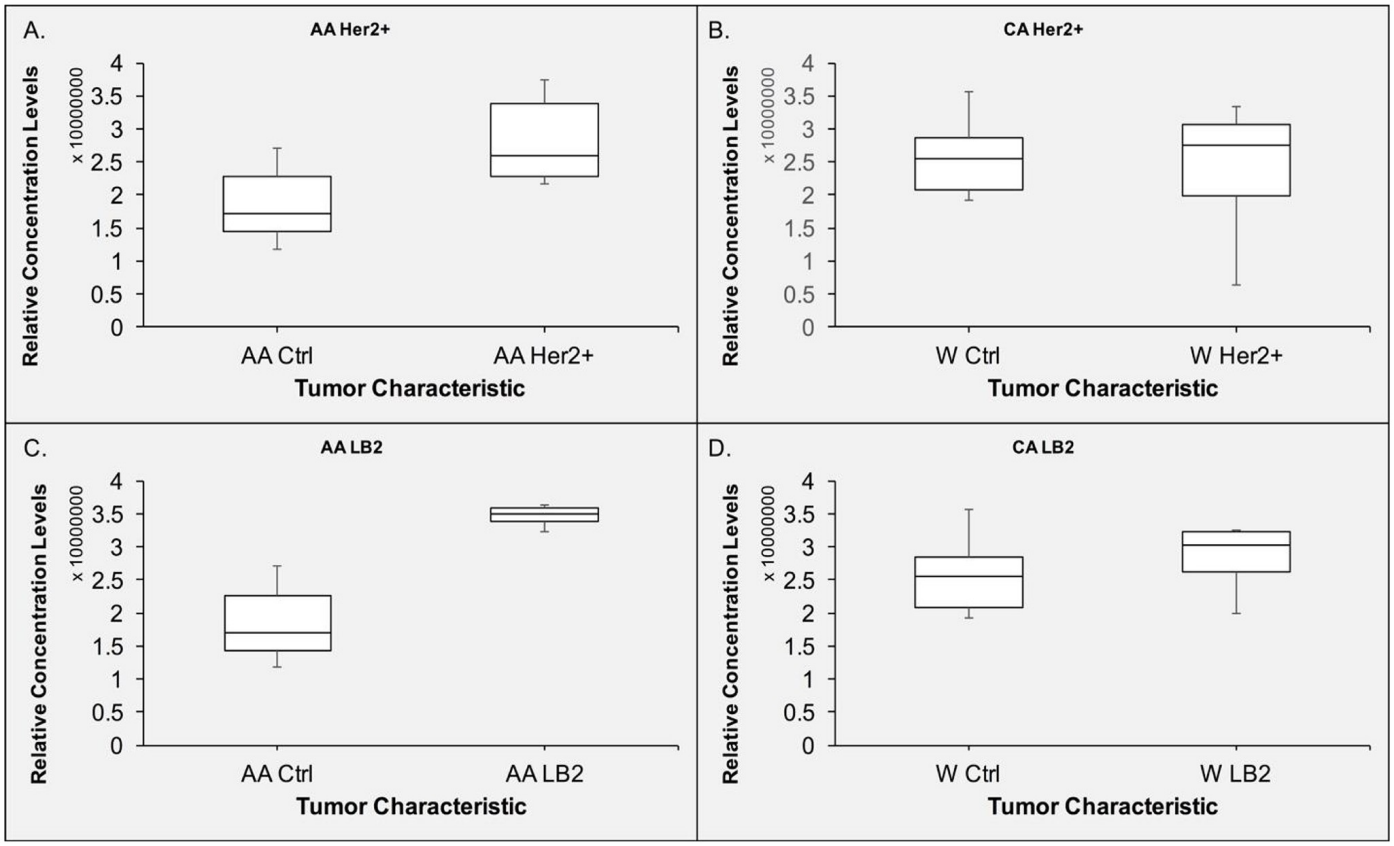
Validation of RPPA data and assessment of racial disparities in serum concentration levels of vitronectin in Her2+ and LB2 tumor subtypes. The data above is validation of data shown in Fig 2, that showed potential racial disparities in tumor subtypes in vitronectin concentration levels of serum. **A**. Displays the relative concentration levels of vitronectin in AA Her2+ patients in serum compared with corresponding control samples. **B**. Relative serum concentration levels of vitronectin in WA Her2+ patients and compared with healthy patients **C**. Displays the relative serum concentration levels of vitronectin in AA LB2 patients and control serum. **D**. Displays the relative concentration levels of vitronectin in CA LB2 patients in serum.

To validate if the vitronectin levels were significantly different between control and Her2+ patients and control and LB2 patients both overall and within same ethnicity (Fig 2C and 2E; Table 2), A SimpleWestern experiment was implemented to confirm this relationship. We analyzed both subtypes by ethnicity, and found that AA patients in both Her2+ and LB2 subtype groups had different vitronectin concentration levels compared to the control group (Fig 3A and 3C). However, the WA patients in both subtypes show very minimal differences (Fig 3B–3D), unlike what was seen in Fig 2E.

### Vitronectin levels in recurrent versus non-recurrent breast cancer patients

**Fig 4:**
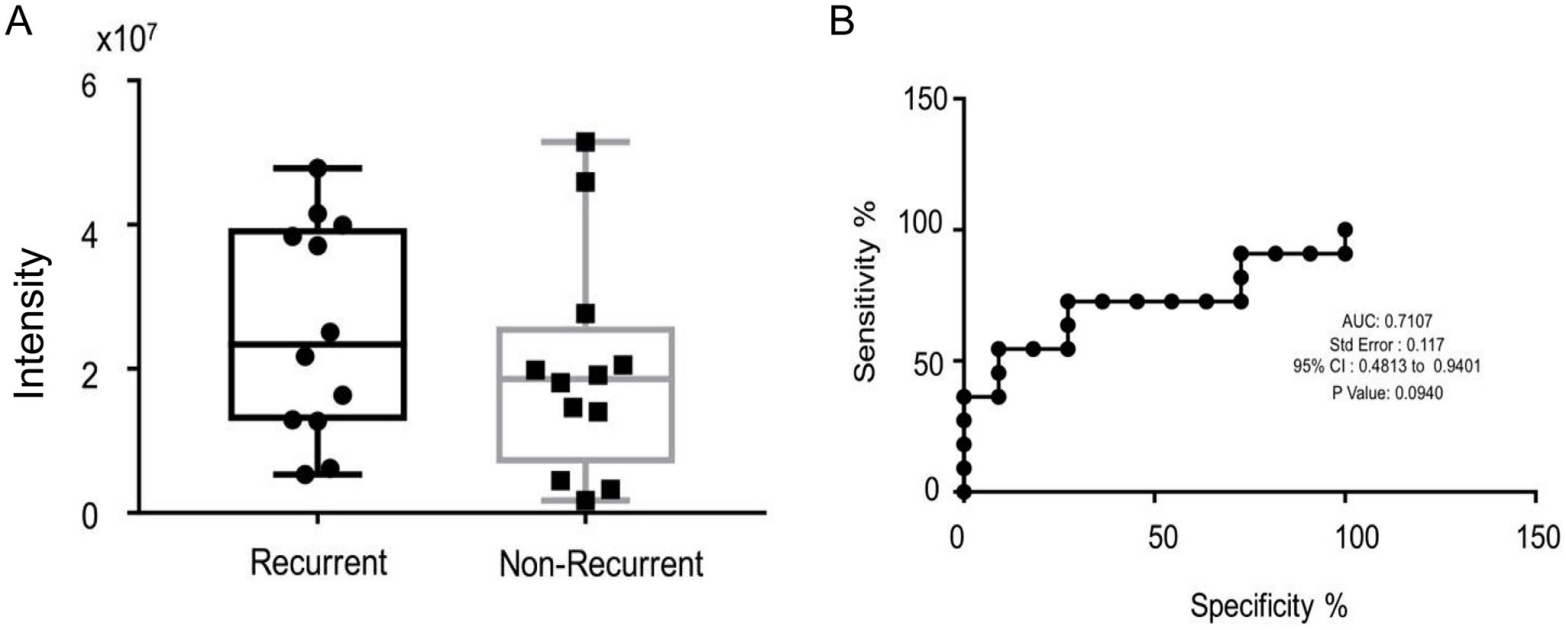
Recurrence and vitronection concentration level in serum. **A**. Overall concentration level of vitronectin in 21 recurrence samples (n = 21) and compared serum vitronectin concentration levels to 21 non-recurrence samples of same IHC sub-groups. **B**. Receiver Operation Characteristic (ROC) Curve and, the statistical evaluation of vitronectin concentration in serum samples between non-recurrence vs recurrence patients. We tested to see if vitronectin could be used as a recurrence biomarkers and, we found that the area under curve (AUC) is 0.7107 with a p-value 0.0940.

Based on our findings from the RPPA analysis, we decided to analyze vitronectin concentration levels in recurrent patients. Out of the 240 serum samples used for RPPA analysis, 21 of these cancer patients showed recurrence after 5–7 years of disease free survival. Next, we performed experiment to compare the vitronectin level of these serum samples with similar sub-typed non-recurrence serum samples (n = 21). Therefore, 21 samples were recurrent patients while the other 21 were samples of non-recurrent patients. We found that there was a difference between vitronectin levels, with recurrent patients having a higher level of vitronectin than non-recurrent patients (Fig 4A). Next, we created a receiver operating characteristic curve (ROC curve) to help us determine if vitronectin concentration levels can be used as a biomarker for recurrence or not. After analysis of the ROC curve, we found that area under the curve (AUC) to be about 0.7107, with a p-value of 0.0940 (Fig 4B).

### Vitronectin concentration levels regulated by PI3K/AKT pathway

**Fig 5:**
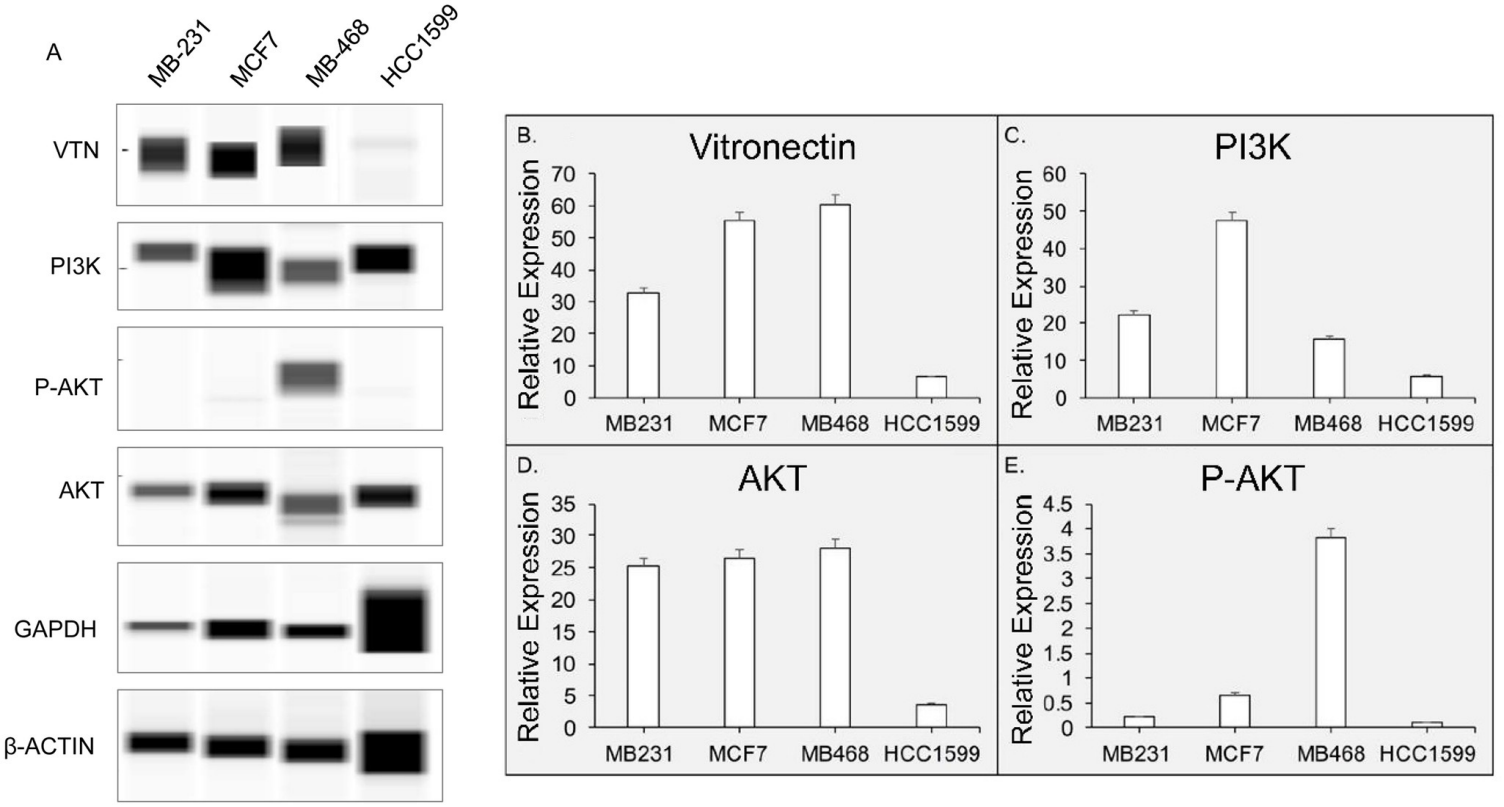
Vitronectin expression is modulated by PI3K/AKT axis. **A**. Displays the pseudo blot extracted from SimpleWestern experiments. There are four cell-lines- MDA-MB-231, MCF7, MDA-MB-468, HCC1599, were used to evaluate the signaling cascade relationship. Different protein expression levels were normalized with an averaged house-keeping GAPDH and β-actin expression **B**. Compares vitronectin concentration levels in four BC cell lines. **C**. PI3K concentration levels in BC same cell-lines. **D**. compares AKT concentration and, **E**. P-AKT concentration levels in the same four BC cell lines.

Next, we analyzed the pathway involved with vitronectin regulation, we analyzed the pathway analysis with the same patient database (METABRIC study) which we had used for vitronectin copy number alteration (CNA) associated survival. We found that vitronectin is associated with PI3K/AKT pathway (S1 and S2 Figs of S1 File). Therefore, we analyzed the proteins that are involved in the same pathway as vitronectin, Phosphoinositide 3-kinases (PI3K), Protein Kinase B (AKT), and Phosphorylated AKT (P-AKT (S473)). The cell-signaling pathway analysis was performed with four different cell lines- MDA-MB-231; MDA-MB-468, MCF-7 and HCC-1599. After running the SimpleWestern, analysis of the vitronectin and other regulating proteins concentration levels in breast cancer cell lines revealed that PI3K and P-AKT regulate vitronectin concentration levels in BC cell lines (Fig 5). A SimpleWestern pseudo-blot was produced to show intensity of signals produced (Fig 5A). The data was then extracted from the Compass Software and plotted after normalization with house-keeping proteins (β-Actin and GAPDH). When comparing vitronectin expression levels, we find that there are MCF7 and MB-468 cell lines have the higher expression compared to MB-231 or HCC1599 cell-lines. More specifically, a 2-fold increase in vitronectin expression levels in MB-468 and MCF-7 cell lines compared to the MB-231 cell line and, a 6-fold increase in MB-468 and MCF-7 cell lines compared to HCC-1599 cell line. However, when looking at P-AKT concentration levels, we find an increase P-AKT concentration levels are only found in MB-468, where MB-468 has a 4-fold increase in P-AKT concentration levels compared to MCF-7, and a 20-fold increase compared to MB-231, and a 40-fold increase compared to HCC-1599. Remarkably, an increase in PI3K concentration levels is only found in MCF-7, where MCF-7 has a 2-fold increase in PI3K compared to MB-231, a 3-fold increase compared to MB-468, and a 9-fold increase compared to HCC-1599.

## Discussion

As mentioned above, BC is the most common cancer in women worldwide, with nearly 2.1 million diagnoses annually. Survival rates vary based on many compounding factors such as age, race, recurrence, and stage. A potential biomarker that can be used to improve diagnosis and then survival rate of BC is vitronectin. Previous studies of vitronectin indicate that it has been linked to various forms of cancers that includes ovarian cancer, prostate cancer, cervical cancer, neuroblastoma, and breast cancer [5, 26–28]. These studies examine VTN expression levels in patients with cancer versus control groups to determine if vitronectin can be used as a biomarker for that cancer. The amino acid sequence that follows the cysteine-rich somatomedin B domain (SMB) sequence is an Arg-Gly-Asp (RGD) peptide sequence that is responsible for the mediating the attachment and spreading of cells to the extracellular matrix by binding to integrin receptors such as integrin αvβ3. Data gathered from the CCLE and TCGA shows VTN is amplified in certain subsets of the population versus others. Patients with amplified VTN were shown to have lower survival rate than those without amplified VTN. We examined the differences between subsets of the population in this study.

Since vitronectin is abundantly found in serum, finding changes in vitronectin concentration in serum, indicates that vitronectin concentration plays a role in that disease. Some studies have shown vitronectin could be used as a potential serum biomarker for BC [29, 30]. These studies focus on serum vitronectin levels in various clinical stages of BC development, and compared them to other biomarkers to determine if vitronectin is an effective serum biomarker for BC patients. In our current study, we focus on how serum vitronectin levels differ in BC patients due to race, recurrence, and IHC characterized different BC subtypes.

The objective of our present study was to determine Vitronectin’s functional role as a non-invasive biomarker that could help with early or late stage diagnoses of BC. Our study differs from previous studies that have considered vitronectin to be a biomarker for BC because we examine vitronectin’s role as racial biomarker, recurrence biomarker, and the mechanistic pathway of vitronectin. After careful examination of our data we found that the results show that there are some differences in vitronectin concentration levels between WA vs AA groups and between recurrent vs non-recurrent groups in WA. Overall, when including all patients, we see that there are differences between Her2+ group vs control and LB2 group vs control (p = 0.004 and p = 0.049). However, after analyzing data by race, we found that there is a significant difference between cancer vs control groups in WA patients only (Fig 2D), whereas there is not a significant difference between cancer and control groups in AA patients. Next, we analyzed both racial groups and compared vitronectin levels in each tumor IHC characterized sub-types.

When comparing serum vitronectin levels across the various tumor IHC characteristics, most appear to have comparable vitronectin levels between the WA group and the AA group. However, some key differences could be found between both groups suggesting that there are racial disparities in vitronectin concentration level and BC. As seen in our validation experiment (Fig 3), both AA LB2 and Her2+ IHC subgroups show higher vitronectin levels than the WA counterparts. Interestingly, TN group shows a significant difference in WA group and approaching significance for AA group in the opposite direction (Fig 2E). These differences in vitronectin levels by race should be further explored to find out if vitronectin is a viable racial biomarker for BC.

Ethnicity also seems to play a role in BC recurrence, as vitronectin concentration levels varied by race between recurrent and non-recurrent patients. WA non-recurrent patients expressed much higher levels of vitronectin compared to their AA counterparts, while recurrent patients from both AA and WA groups expressed similar levels of vitronectin. We found that there was a significant difference in WA non-recurrent groups than in other groups (Fig 2F).

In this study, we also analyzed the serum concentration levels of vitronectin and related proteins in various BC cell lines. We analyzed which genes could potentially be involved in the vitronectin pathway, and found genes that were involved in overexpression of vitronectin. Using the METABRIC database as previously mentioned we found that PI3K was a gene that was involved in the pathway. To confirm the relationship between the PI3K and VTN, we performed the SimpleWestern analysis with BC cell lines, MDA-MB-231, MDA-MB-468, MCF-7, and HCC1599, comparing serum concentrations levels of VTN, PI3K, AKT, and P-AKT. We found that the VTN levels increased in certain BC cell lines such as MB-468 and MCF-7. While, the concentration levels of PI3K and AKT correlate with the concentration levels of serum vitronectin, suggesting that the vitronectin is regulated by the PI3K/AKT mechanistic pathway. The PI3K/AKT pathway is a crucial player in the development of cancer. AKT, regulates diverse cellular functions from metabolism, growth, proliferation and more [31, 32]. PI3K activates phosphorylates that activated AKT. The dysregulation of this pathway often leads to cancer, such as breast cancer. Previous studies have PI3K/AKT pathway involved with BC progression [33, 34]. The possible involvement of Vitronectin with the PI3K/AKT pathway suggests that there is another crucial player involved BC progression. PI3K/AKT has been shown to activate by surface integrins that leads to metastasis of tumors, as seen in gastric carcinoma cells [35]. Integrin αvβ3, could be the crucial link for fully understanding the mechanistic pathway that vitronectin uses in BC.

For future studies, we hope to evaluate how Integrin αvβ3 serum levels impact vitronectin levels and BC tumor formation. Previous studies have mentioned how Integrin αvβ3 works with vitronectin to promote cell adhesion and metastasis of tumors. Since vitronectin prominently accumulates in the ECM of tumor cells of various carcinomas, especially breast cancer cells [36]. This then allows vitronectin to bind with Integrin αvβ3, and then enhance angiogenesis and neovascularization of tissues. A previous study compared the relationship between Integrin αvβ3 and vitronectin, and considered if the two can be used together or separately as a biomarker for higher stages of breast cancer [19]. One potential study we can conduct would be to look at the racial and recurrence disparities of integrin αvβ3 serum concentration levels. We can then potentially look at integrin αvβ3 levels in serum and compare them to proteins in PI3K/AKT pathway alongside vitronectin. For the future, we also hope to use larger sample sizes to get data that is more statistically significant.

## Supporting information

S1 File (DOCX)

## Abbreviations

AA: African American
BC: Breast Cancer
Her2: human epidermal growth factor receptor 2 subtype
LA: Luminal A subtype
LB1: Luminal B1 subtype
LB2: Luminal B2 subtype
RPPA: Reverse Phase Protein Array
TN: triple-negative subtype
WA: White American or Caucasian American (CA)
