# Supplementary material for: Functional role of vitronectin in breast cancer
Source: PLoS One. 2020 Nov 19;15(11):e0242141. doi: 10.1371/journal.pone.0242141 (PMC7676670; doi:10.1371/journal.pone.0242141)

Supplementary documents:


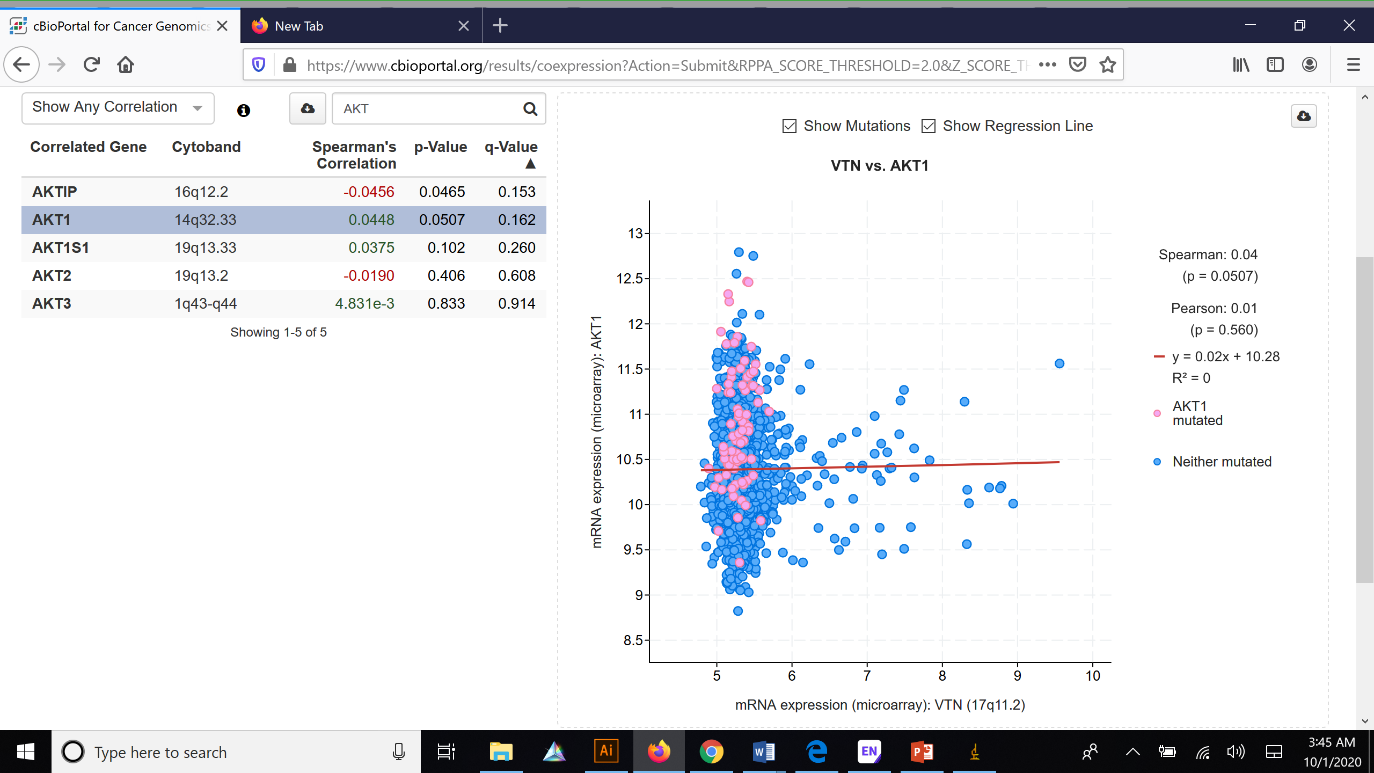

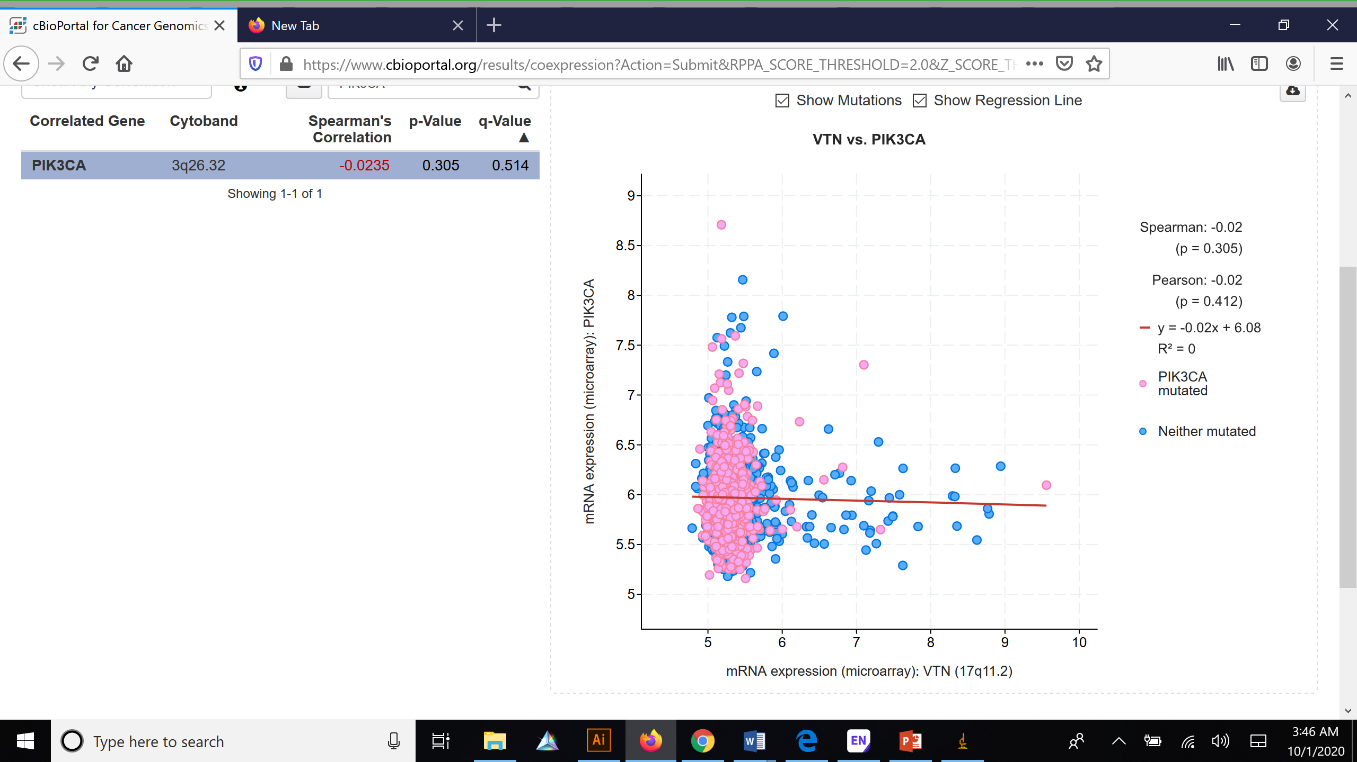


**S1 Fig** **S2 Fig 2**

**S1 and S2 Figs**. Correlation studies for gene of interest VTN, AKT1 & PIK3CA. The Spearman and Person coefficient were calculated for Breast Cancer (METABRIC, n= 2173 patients/samples Nature 2012 (22) & Nat Commun 2016 (23)) studies.

Supporting material- whole blot picture (for Fig 5A). Following images are the full pseudo-blot images obtained from WES (protein simple; capillary electrophoresis followed by primary and secondary antibodies staining; details in materials and methods section) instrument analyzed with Compass software. Four different BrCa cell lines-- MB-231; MCF7, MB-468 and HCC 1599 were stained with six different antibodies as follows:


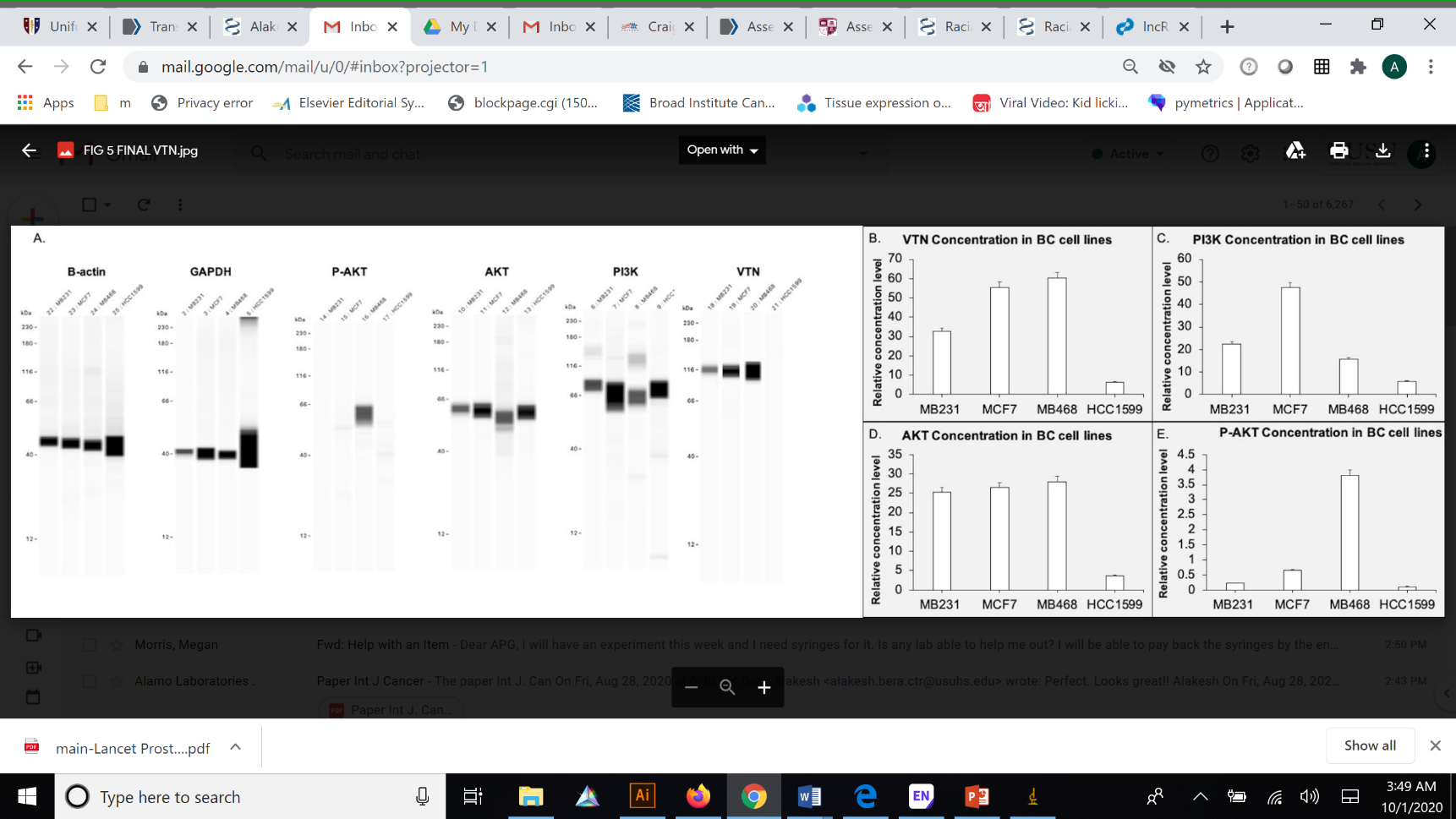

Supplement: S1 File — (DOCX) [file pone.0242141.s001.docx]
